# Characterization of the complete mitochondrial genome of the maize weevil, *Sitophilus zeamais* (Insecta: Coleoptera: Curculionidae) from Guizhou province

**DOI:** 10.1080/23802359.2020.1731354

**Published:** 2020-02-28

**Authors:** Xin Zhou

**Affiliations:** College of Mathematics and Information Science, Guiyang University, Guiyang, China

**Keywords:** *Sitophilus zeamais*, the maize weevil, Curculionidae, mitochondrial genome, stored-product insect

## Abstract

The complete mitochondrial genome of the maize weevil *Sitophilus zeamais* (GenBank accession number: MN905575) from Guizhou Province consists of a circular DNA molecule of 18,421 bp (with 75.69% A + T content), which is longer by 316 bp than that of the mitogenome of *S. zeamais* from Nigeria. The mitogenome comprises 13 protein-coding, 22 tRNA, and two rDNA genes. The protein-coding genes have typical ATN (Met) initiation codons and are terminated by typical TAN stop codons.

The maize weevil, *Sitophilus zeamais* threaten the food security worldwide. There is only one other mitochondrial sequence of *S. zeamais* (GenBank: NC_030764.1) from Nigeria (Ojo et al. 2016). Here, we report the characterization and phylogenetic studies of the complete mitogenome of *S. zeamais*, which obtained by Yu Bai from Guiyang University (E106.7748235166073°, N26.556384479324556°), Guiyang City, Guizhou Province, China, on 25 August 2019, to obtain more comprehensive biological insight into the population genetics of *S. zeamais*. Samples of adult *S. zeamais* were deposited by Yu Bai in the animal specimen room of Guiyang University with Specimen Accession Number: GYU-20190825-001. Genomic DNA was isolated and fragmented to build a genomic library of the approximate insert size 350 bp that was sequenced (paired-end 2 × 150 bp) using an Illumina HiSeq 4000 (Illumina, Inc., San Diego, CA, USA). We obtained 75,548,982 reads of high-quality, clean data cleaned by cutadapt version 1.9.1 (Martin 2011). The mitochondrial genome was assembled *de novo* with Velvet 1.2.10 (Zerbino and Birney 2008), gapfilled with SSPACE 3.0 (Boetzer et al. 2011), and GapFiller 1.1(Boetzer and Pirovano 2012).

The mitogenome of *S. zeamais* consists of a 18,421 bp circular DNA molecule (GenBank: MN905575), with 39.74% A, 35.95% T, 15.48% C, and 8.83% G, which has an A/T bias (75.69% A + T content) and is longer by 316 bp than the *S. zeamais* mitogenome of the Nigeria population. The AT- and GC-skews of the major strands of the mitogenome were calculated to be approximately 0.0337 and 0.2736, respectively. The length of the A/T-rich region in the mitogenome is 2839 bp, with 80.13% A + T content, and is located between the srRNA and tRNA-Ile. The order and orientation of the functional areas of the *S. zeamais* mitogenome are identical to those in the *Tenebrio obscurus*, *Zophobas atratus*, and *Oryzaephilus surinamensis* mitogenome (Bai et al. 2018, 2019; Liang et al. 2019). The mitogenome of *S. zeamais* contained 13 protein-coding genes (PCGs), 22 tRNA, and two rRNA genes. All 13 PCGs had typical ATN (Met) start codons: *nad3* and *nad1* for ATA; *nad5*, *atp8*, *cox2*, *cox1*, *nad2*, and *nad6* for ATT; *nad4*, *cox3*, *atp6, cob*, and *nad4l* for ATG. All 13 PCGs had typical TAN stop codons: *cox3*, *atp6*, *cox1*, *nad2*, *nad4l*, *cob*, and *nad6* for TAA; *nad5*, *atp8*, *cox2*, and *nad1* for TAG; *nad3* and *nad4* for an incomplete stop codon consisting of a T− that is completed by the addition of 3′ A nucleotides to the resultant mRNA. The 22 tRNA genes were interspersed throughout the coding region and ranged from 63 (trnC) to 71 bp (trnK) in length. lrRNA and srRNA were 1296 and 780 bp long, respectively.

To validate the phylogenetic position of *S. zeamais*, the mitogenome DNA sequences from 11 species of Curculionidae were used to construct a phylogenetic tree by the Maximum-Likelihood method using the MEGA 7 (Kumar et al. 2016) (Figure 1). In conclusion, our study provides information of the mitogenome of *S. zeamais*, which will be useful for molecular identification and phylogenetic studies.

**Figure 1.**
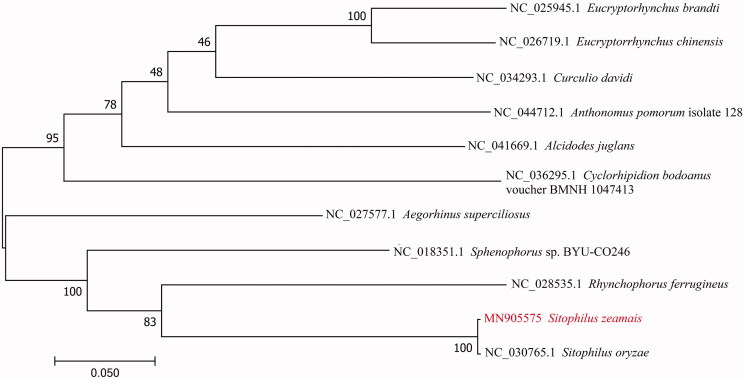
The Maximum-Likelihood phylogenetic tree of *S. zeamais* and other 10 beetles of Curculionidae based on the DNA sequences of mitogenome.
